# A Genetic Variant of miR-34a Contributes to Susceptibility of Ischemic Stroke Among Chinese Population

**DOI:** 10.3389/fphys.2019.00432

**Published:** 2019-04-24

**Authors:** Gui-Jiang Wei, Ming-Qing Yuan, Li-He Jiang, Yu-Lan Lu, Chun-Hong Liu, Hong-Cheng Luo, Hua-Tuo Huang, Zong-Quan Qi, Ye-Sheng Wei

**Affiliations:** ^1^Department of Cell Biology, Medical College of Guangxi University, Nanning, China; ^2^Department of Medical Laboratory, Affiliated Hospital of Guilin Medical University, Guilin, China; ^3^Department of Medical Laboratory, Affiliated Hospital of Youjiang Medical University for Nationalities, Baise, China

**Keywords:** ischemic stroke, miR-34a, single nucleotide polymorphism, genotype, risk

## Abstract

miRNAs are small non-coding RNAs modulating gene expression, and variants in miRNA genes are involved in the pathogenesis of ischemic stroke (IS). However, the effect of miR-34a polymorphisms on IS susceptibility has rarely been reported. In the present study, we investigated the association between rs12128240, rs2666433, and rs6577555 of the miR-34a gene and IS susceptibility. Snapshot assay was used to detect miR-34a polymorphisms in 548 IS patients and 560 controls. Relative expression of miR-34a was measured by quantitative real-time PCR. We found that rs2666433 was associated with a significantly increased risk of IS (AA vs. GG: OR = 1.61, 95% CI = 1.05–2.52, *P* = 0.031; AA vs. GG+GA: OR = 1.58, 95% CI = 1.05–2.45, *P* = 0.026). For the IS subtypes, rs2666433 was associated with large artery atherosclerosis (AA vs. GG: OR = 2.09, 95% CI = 1.16–3.51, *P* = 0.007; AA vs. GG+GA: OR = 2.02, 95% CI = 1.15–3.33, *P* = 0.007; A vs. G: OR = 1.36, 95% CI = 1.07–1.81, *P* = 0.021). Additionally, the level of miR-34a was significantly up-regulated in IS patients compared to the controls (*P* < 0.001), and patients with rs2666433 AA genotype had a higher level of miR-34a than those with GG+GA genotypes (*P* < 0.001). Furthermore, increased level of homocysteine was observed in IS patients compared to the controls (*P* < 0.001), especially in patients carrying the rs2666433AA genotype compared to those carrying the rs2666433 GG+GA genotypes (*P* < 0.001). However, no significant association between rs12128240 or rs6577555 and IS was found. Collectively, our study found the association between miR-34a polymorphisms and the risk of IS among the Chinese population. The results may provide an explanation for etiology of IS and a potential biomarker or therapeutic target for IS.

**HIGHLIGHTS**
- MiR-34a rs2666433 polymorphism was associated with an increased risk of ischemic stroke.
- The level of miR-34a was significantly up-regulated in ischemic stroke patients compared with controls, and patients with rs2666433 AA genotype had a higher level miR-34a than those with GG+GA genotypes.
- Furthermore, increased level of homocysteine was showed in IS patients compared to controls, and in patients carrying the rs2666433AA compared to those carrying the rs2666433 GG+GA.

## Introduction

Stroke is a leading cause of mortality among the elderly population in the world (Adeloye, 2014; Kim, 2014; Robert and Zamzami, 2014). It can be divided into hemorrhagic and ischemic strokes (IS), with the latter being the most common form of stroke, accounting for 42–79% of all strokes (Zhang et al., 2003). In China, because of its high incidence, IS has exceeded the incidence of heart diseases and cancers, bringing great economic and health burdens to families and society (Liu et al., 2011). Atherosclerosis is an inflammatory reaction that involves aggregation of leukocytes, vascular smooth muscle cell and endothelial senescence (Libby, 2002). The etiology of atherosclerosis is complex but generally involves the formation of atherosclerotic plaques. Evidence showed cardiovascular obstruction causes coronary artery disease and cerebrovascular obstruction leads to IS (Weber and Noels, 2011). Multiple factors, including diabetes mellitus, hypertension, smoking, hyperhomocysteinemia and hyperlipidemia, are associated with a higher risk of IS (Tuhrim, 2000). Nevertheless, these risk factors just clarify some parts of IS pathogenesis. Clinical and epidemiological studies suggest genetic factors play an important role in IS pathogenesis (Jerrard et al., 2003).

MiRNAs are small non-coding RNAs which bind to the 3′-untranslated region (3′-UTR) of target messenger RNAs (mRNAs), and then promote mRNAs degradation or inhibit translation (Bhaskaran and Mohan, 2014). Many studies have shown that they play important roles in various diseases such as neurodegenerative disorders (Karnati et al., 2015), cancer (Wang et al., 2017), cardiovascular diseases (Srinivasan and Das, 2015) and stroke (Khoshnam et al., 2017). The miR-34a gene, located at the chromosome 1p36 locus, is a modulator of the vascular smooth muscle cell and endothelial senescence (Badi et al., 2015; Ito et al., 2010). The up-regulated expression of miR-34a was observed in both atherosclerosis and IS (Raitoharju et al., 2011; Liang and Lou, 2016), and inhibition of miR-34a prevents endothelial cell apoptosis in the setting of atherosclerosis (Li et al., 2018). These outcomes suggest that miR-34a is a potential IS biomarker or a new target for IS therapy.

Single nucleotide polymorphisms (SNPs) are the most common variations at specific base positions of the human genome. The expression of miRNAs can be affected by SNPs locating in the coding genes of miRNAs, and subsequently change a variety of biological functions (Kim et al., 2016). Previous works have shown that the susceptibility of individuals to multiple human diseases may be modulated by SNPs of the miR-34a gene such as osteosarcoma (Lv et al., 2014), colon cancer (Jiang et al., 2017), type 2 diabetes (Sun et al., 2018) and IS (Choi et al., 2016). However, the association between miR-34a SNPs and IS susceptibility has not been reported in the Chinese population. To examine the association between miR-34a SNPs and IS susceptibility, we performed a case-control study. Three SNPs of the miR-34a gene, namely rs12128240, rs2666433 and rs6577555, were selected based on location, minor allele frequency (MAF) and previous reports. Meanwhile, we also assessed the impact of miR-34a polymorphisms on the expression of miR-34a and other IS-related factors.

## Materials and Methods

### Study Population

Five hundred and forty-eight patients in this study were recruited from the Department of Neurology, Affiliated Hospital of Youjiang Medical University for Nationalities, Guangxi, China in the period from January 2017 to December 2017. Based on clinical symptoms, neurological examination, magnetic resonance imaging or/and computed tomography, an IS patient was diagnosed by two experienced clinical neurologists. According to Trial of ORG 10172 in Acute Stroke Treatment (TOAST), only patients with subtypes of small artery occlusion (SAO) and large artery atherosclerosis (LAA) were included in this study (Adams et al., 1993). Exclusion criteria were listed below: recurrent IS, hemorrhagic stroke, tumorous, traumatic, infectious and cardiogenic cerebrovascular diseases, cerebrovascular malformation and severe mental disorder. To check the neurological status of the patients, National Institutes of Health Stroke Scale (NIHSS) on admission was used to scale IS severity (Tilley et al., 1996). To determine the relationship between the miR-34a gene polymorphisms and IS severity, IS patients were divided into two subgroups according to the NIHSS score (<6 and ≥6) (O’Donnell et al., 2010).

Five hundred and sixty gender and age-matched controls were recruited from the Health Examination Department of Affiliated Hospital of Youjiang Medical University for Nationalities, Guangxi, China in the period from January 2017 to December 2017. Exclusion criteria were listed below: stroke history, cancer, informatory, genetic, autoimmune, neurological or cardiogenic diseases. All the patients and controls came from the Guangxi Zhuang Autonomous Region of China.

Clinical information for patients and controls comprising age, gender, hypertension, diabetes mellitus, smoking and stroke history were recorded on admission or receiving health examination, respectively. Hypertension was defined as receiving anti-hypertensive treatment currently or having a mean diastolic blood pressure ≥ 90 mm Hg or/and a systolic blood pressure ≥ 140 mm Hg. Smokers referred to those smoking daily. Blood samples of controls were collected during health examination, and those of patients were collected within 24 h after onset of symptoms on admission. Total cholesterol (TCH), homocysteine, triglyceride, low-density lipoprotein cholesterol (LDL-C), high-density lipoprotein cholesterol (HDL-C), very low-density lipoprotein cholesterol (VLDL-C) and high sensitivity C-reactive protein (hsCRP) were determined.

The study was approved by the Ethics Committee of Youjiang Medical University for Nationalities and conformed to the guidelines set forth by the Declaration of Helsinki. Meanwhile, written informed consent was obtained from all patients and controls prior to entering the study.

### SNPs Selection and Genotyping

The criteria to select the SNPs in this study include: (I) Minor allele frequency is greater than 10% in the southern Han Chinese population; (II) The SNPs should locate in the regulatory regions of the miR-34a gene; (III) The SNPs have been reported by previous references. According to previous reports (Kim et al., 2012), 555 SNPs were found by searching upstream 2 kb and downstream 500 b of the miR-34a gene in the National Center for Biotechnology Information SNP database^1^. Three SNPs (rs2666433/NC_000001.11:g.9153118A>G and rs6577555/NC_000001.11:g.9152228A>C in the promoter; rs12128240/NC_000001.11:g.9151244C>T in the 3′-flanking region) with MAF greater than 10% in southern Han Chinese population were selected.

DNA extraction kits (Yaneng, China) were used to extract genomic DNA from whole blood samples and the genomic DNA was stored at –80°C. Primers for genotyping SNPs of the miR-34a gene were designed by Primer Premier 6 and synthesized by Shanghai Sangon Corporation. Primers for genotyping SNPs of the miR-34a gene are listed in Supplementary Table 1. After amplifying the miR-34a gene by polymerase chain reaction, the rs12128240, rs2666433 and rs6577555 polymorphisms were genotyped by Snapshot assay. Finally, Sanger DNA sequencing was used for verifying genotyping results of Snapshot assay.

### Quantitative Analysis of miR-34a

The analysis was conducted on a subgroup of 79 IS patients and 79 controls, who were chosen according to the results of genotyping. Among the 79 IS patients, 39 patients had AA genotype and 40 patients had GG+GA genotypes. There was a similar balance in the controls. Total RNA was extracted from 200 μL plasma of controls and patients following the instruction of RNA extraction kit (Qiagen, Germany). Synthetic cel-miR-39 was used for normalization of sample variation based on the previous studies (Hauser et al., 2012; Wang et al., 2018), and validated in our previous study. The concentration of RNA was determined by Nanodrop 2000 Spectrophotometer (Thermo Fisher Scientific, United States). Reverse transcription kit (Ribobio, China) was used to transcribe 100 ng of total RNA into complementary DNA. The products of reverse transcription were stored at –80°C. Quantitative real-time PCR was performed by Mir-X miRNA qRT-PCR SYBR Kit (Takara, China), on Applied Biosystems 7900HT system (ABI, United States) and followed these steps: 95°C for 10 s, 40 cycles of 95°C for 5 s and 60°C for 20 s. According to the result of quantitative real-time PCR for miR-148b conducted previously in our laboratory, miR-148b was chosen as a negative control in this study. The primers for miR-34a/39/148b were designed and synthesized by Ribobio Corporation. Reactions were performed in triplicate. Relative expression levels of miR-34a and miR-148b were calculated by 2^Ct(cel-miR-39)-Ct(miR-34a)^ and 2^Ct(cel-miR-39)-Ct(miR-148b)^, respectively.

### Statistical Analysis

Statistical analysis was performed by Statistical Package for Social Science (version 17.0). Hardy-Weinberg equilibrium testing and Categorical variables comparison were proceeded by two-sided chi-square test. The normality of continuous variables was assessed by the Shapiro–Wilk test. Continuous variables were analyzed by the Mann–Whitney *U* test or the Student’s *t*-test. Logistic regression was used for evaluating the association between miR-34a polymorphisms and IS risk, and odds ratio (OR), 95% confidence interval (CI) and *P*-values were adjusted by gender, age, smoker, diabetes and hypertension. The Benjamini–Hochberg(B-H) method was applied to control the false discovery rate. Genotypes and alleles distribution of rs2666433 in different populations were obtained from the website below^2^. Online bioinformatics software was used to predict the potential targets of miR-34a^3^. An online tool SHEsis50 was used for Haplotype analysis (SNPs order: rs12128240-rs2666433-rs6577555). *P* < 0.05 indicated statistical significance. The power calculation was performed by the GAS Power Calculator. Because the MAF of rs6577555 was lower than rs12128240 and rs2666433, the power calculation was performed according to rs6577555. Under the parameters of disease prevalence = 0.016 (Jiang et al., 2017), MAF = 0.20, significance level = 0.05, OR = 1.5 and sample size (cases = 548, controls = 560), we had 95% statistical power to identify a convincing association between SNPs and IS risk.

## Results

### Clinical Features

The clinical features of controls and IS patients are shown in Table 1. Smoking, hypertension, diabetes and high levels of TCH, homocysteine, hsCRP, LDL-C were more common in the IS patients than the controls (*P* < 0.001). In contrast, the level of HDL-C in the IS patients was significantly lower than that in the controls (*P* < 0.001). Furthermore, as for gender, age, triglyceride and VLDL-C, there were no significant differences between IS patients and the controls (*P* = 0.739, *P* = 0.284, *P* = 0.432 and *P* = 0.498, respectively). As shown in Supplementary Table 3, as for gender, age and smoker, there were no significant differences between the NHISS < 6 patients and the NHISS ≥ 6 patients (*P* = 0.541, *P* = 0.192 and *P* = 0.130, respectively). However, as for hypertension and diabetes mellitus, there were significant differences between the NHISS < 6 patients and the NHISS ≥ 6 patients (*P* = 0.011 and *P* = 0.008, respectively).

**Table 1:** Clinical characteristics of the study population.

Variables	Controls (*n* = 560)	IS patients (*n* = 548)	*P*^†^
Gender (M/F)	379/181	376/172	0.739
Age, years (mean±SD)	59.88±10.12	59.34±9.25	0.284
Smoker, *n* (%)	83(14.8)	158(28.8)	**<0.001**
Hypertension, *n* (%)	146(25.5)	291(53.1)	**<0.001**
Diabetes mellitus, *n* (%)	57(10.1)	158(28.8)	**<0.001**
TCH, mmol/L	4.97±1.16	5.55±1.08	**<0.001**
TG, mmol/L	1.56±0.72	1.61±0.84	0.432
HDL-C, mmol/L	1.69±0.55	1.39±0.44	**<0.001**
LDL-C, mmol/L	2.17±1.08	2.88±1.23	**<0.001**
VLDL-C, mmol/L	0.79±0.67	0.77±0.63	0.498
Hcy, μmol/L	8.92±2.43	12.14±2.61	**<0.001**
hsCRP, mg/L	2.99±1.25	5.37±1.50	**<0.001**


*IS: ischemic stroke; SD: standard deviation; M: male; F: female; TCH: total cholesterol; TG: triglyceride; HDL-C: high-density lipoprotein cholesterol; LDL-C: low-density lipoprotein cholesterol; VLDL-C: very low-density lipoprotein cholesterol; Hcy: homocysteine; hsCRP: high sensitivity C-reactive protein. ^†^Two-sided chi-square test or student’s t-test. *P*< 0.05 were indicated in bold font.*

### Association Between the miR-34a Polymorphisms and the Risk of IS

Sequencing diagrams of rs12128240, rs2666433, and rs6577555 are shown in Supplementary Figure 1. The association between the rs12128240, rs2666433 or rs6577555 polymorphism and IS risk is shown in Table 2. The genotypes of rs12128240, rs2666433, and rs6577555 in both controls (*P* = 0.434, *P* = 0.992, and *P* = 0.975, respectively) and cases (*P* = 0.491, *P* = 0.318 and *P* = 0.866, respectively) were in Hardy-Weinberg equilibrium. We found that the rs2666433 AA genotype was associated with a significantly increased IS risk compared with the GG genotype (AA vs. GG: OR = 1.61, 95% CI = 1.05–2.52, *P* = 0.031). Similarly, we also observed a significantly increased risk of IS in AA vs. GG+GA model analysis (AA vs. GG+GA: OR = 1.58, 95% CI = 1.05–2.45, *P* = 0.026). However, the statistical significance was lost after correcting the *P*-values by B-H method. Additionally, no significant association between the rs12128240 or rs6577555 and the risk of IS was observed.

**Table 2:** Association between the miR-34a polymorphisms and risk of IS.

SNPs	Controls (*n* = 560)	IS patients (*n* = 548)	OR (95% CI)^†^	*P*^†^, *P^BH^*
**rs12128240**
CC	334 (59.6)	329 (60.0)	1.00(ref)	
CT	187(33.4)	182(33.2)	0.94(0.57–1.66)	0.874, 0.874
TT	39(7.0)	37(6.8)	0.96(0.56–1.79)	0.826, 0.826
TT+CT vs. CC			0.97(0.74–1.50)	0.832, 0.866
TT vs. CC+CT			1.17(0.66–1.82)	0.854, 0.854
C	855(76.3)	840(76.6)	1.00(ref)	
T	265(23.7)	256(23.4)	0.98(0.78–1.44)	0.808,0.808
**rs2666433**
GG	286(51.1)	265(48.4)	1.00(ref)	
GA	228(40.7)	216(39.4)	1.05(0.77–1.39)	0.868, 0.874
AA	46(8.2)	67(12.2)	1.61(1.05–2.52)	**0.031**, 0.093
AA+GA vs. GG			1.18(0.86–1.51)	0.368, 0.866
AA vs. GG+GA			1.58(1.05–2.45)	**0.026**, 0.078
G	800(71.4)	746(68.1)	1.00(ref)	
A	320(28.6)	350(31.9)	1.23(0.94–1.52)	0.086, 0.258
**rs6577555**
CC	353(63.0)	344(62.8)	1.00(ref)	
CA	188(33.6)	179(32.7)	0.95(0.71–1.51)	0.803, 0.874
AA	19(3.4)	25(4.5)	1.30(0.65-2.92)	0.301, 0.451
AA+CA vs. CC			1.08(0.74–1.61)	0.866, 0.866
AA vs. CC+CA			0.86(0.53–1.78)	0.347, 0.520
C	894(79.8)	867(79.1)	1.00(ref)	
A	226(20.2)	229(20.9)	1.10(0.83–1.36)	0.629, 0.808


*Ref: reference; OR: odds ratio; CI: confidence interval. ^†^Adjusted by gender, age, smoker, diabetes and hypertension. ^*BH*^Corrected by B-H method. *P* < 0.05 were indicated in bold font.*

### Association Between the miR-34a Polymorphisms and LAA or SAO Subtypes of IS

The association between the miR-34a polymorphisms and LAA or SAO subtypes of IS was investigated according to the TOAST classification. As shown in Table 3, AA genotype of rs2666433 was associated with a significantly increased risk of IS LAA subtype compared with GG genotype, and this association remained significant in AA vs. GG+GA model analysis (AA vs. GG+GA: OR = 2.02, 95% CI = 1.15–3.33, *P* = 0.007). Notably, the statistical significance remained after correcting the *P*-values by B-H method. Additionally, A allele of rs2666433 was also associated with a significantly increased risk of IS LAA subtype compared with G allele (AA vs. GG: OR = 2.09, 95% CI = 1.16–3.51, *P* = 0.007; A vs. G: OR = 1.36, 95% CI = 1.07–1.81, *P* = 0.021). In contrast, the rs2666433 was not associated with SAO. Meanwhile, the rs12128240 and rs6577555 were not associated with LAA and SAO. The clinical features of LAA and SAO subgroups are shown in Supplementary Table 2.

**Table 3:** Association of miR-34a polymorphisms with LAA and SAO subtypes of IS.

SNPs	Controls (*n* = 560)	LAA (*n* = 207)	OR (95% CI)^†^*,P*^†^*,P^BH^*	SAO (*n* = 341)	OR (95% CI)^†^*,P*^†^*,P^BH^*
**rs12128240**
CC	334(59.6)	124(59.9)	1.00(ref)	205(60.1)	1.00(ref)
CT	187(33.4)	70(33.8)	1.02(0.74–1.50),0.952,0.952	112(32.8)	0.98(0.68-1.46),0.863,0.912
TT	39(7.0)	13(6.3)	0.93(0.44–1.94),0.765,0.765	24(7.1)	1.02(0.59–2.11),0.981,0.981
TT+CT vs. CC			0.99(0.69-1.48),0.942,0.942		0.99(0.65–1.38),0.889,0.996
TT vs. CC+CT			0.93(0.51-1.69), 0.741,0.751		1.01(0.58–1.82),0.965,0.965
C	855(76.3)	318(76.8)	1.00(ref)	522(76.5)	1.00(ref)
T	265(23.7)	96(23.2)	0.99(0.73-1.37),0.852,0.852	160(23.5)	0.99(0.76–1.29),0.921,0.921
**rs2666433**
GG	286(51.1)	94(45.4)	1.00(ref)	171(50.1)	1.00(ref)
GA	228(40.7)	82(39.7)	1.12(0.74–1.67),0.609,0.952	134(39.3)	0.99(0.70–1.48),0.912,0.912
AA	46(8.2)	31(14.9)	**2.09(1.16**-**3.51),0.006,0.018**	36(10.6)	1.30(0.79–2.24),0.263,0.394
AA+GA vs. GG			1.30(0.91–1.89), 0.175,0.018		1.09(0.79–1.52),0.760, 0.996
AA vs. GG+GA			**2.02(1.15**-**3.33), 0.007,0.021**		1.41(0.81–2.36),0.231,0.367
G	800(71.4)	270(65.2)	1.00(ref)	476(69.8)	1.00(ref)
A	320(28.6)	144(34.8)	**1.36(1.07**-**1.81),0.021,**0.063	206(30.2)	1.13(0.88–1.39),0.463,0.921
**rs6577555**
CC	353(63.0)	129(62.3)	1.00(ref)	215(63.0)	1.00(ref)
CA	188(33.6)	70(33.8)	1.04(0.75–1.53),0.924,0.952	109(32.0)	0.97(0.68–1.33),0.749,0.912
AA	19(3.4)	8(3.9)	1.18(0.56–2.77),0.742,0.765	17(5.0)	1.57(0.73–3.17),0.256,0.394
AA+CA vs. CC			1.07(0.81–1.58), 0.866,0.942		1.02(0.79–1.42),0.996,0.996
AA vs. CC+CA			1.53(0.50–2.94),0.751,0.751		1.58(0.77–3.02),0.245,0.367
C	894(79.8)	328(79.2)	1.00(ref)	539(79.0)	1.00(ref)
A	226(20.2)	86(20.8)	1.07(0.82–1.41),0.803, 0.852	143(31.0)	1.08(0.81–1.45),0.693,0.921


*LAA: large artery atherosclerosis; SAO: small artery occlusion. ^†^Adjusted by gender, age, smoker, diabetes and hypertension. ^*BH*^Corrected by B-H method. *P* < 0.05 were indicated in bold font.*

### Association Between the miR-34a Polymorphisms and the NIHSS Score

To analyze the association between the miR-34a polymorphisms and IS severity, we further investigated the association between the rs12128240, rs2666433 or rs6577555 and the NIHSS score on admission. As shown in Supplementary Table 3, of the 548 patients, 312 individuals were classified as having a mild stroke (NHISS < 6) and 236 as having a severe stroke (NHISS ≥ 6). However, as shown in Table 4, we did not find a significant association between the miR-34a polymorphisms and IS severity (*P* > 0.05).

**Table 4:** Association between the miR-34a polymorphisms and the NIHSS score.

SNPs	NHISS < 6 (*n* = 312)	NHISS ≥ 6 (*n* = 236)	OR (95% CI)^†^	*P*^†^
**rs12128240**
CC	180(57.7)	149(63.1)	1.00(ref)	
CT	109(34.9)	73(31.0)	0.86(0.52–1.33)	0.261
TT	23(7.4)	14(5.9)	0.79(0.30–1.69)	0.383
TT+CT vs. CC			0.83(0.52–1.21)	0.201
TT vs. CC+CT			0.82(0.36–1.72)	0.514
C	469(75.2)	371(78.6)	1.00(ref)	
T	155(24.8)	101(21.4)	0.87(0.58–1.25)	0.189
**rs2666433**
GG	147(47.1)	118(50.0)	1.00(ref)	
GA	133(42.6)	83(35.2)	0.80(0.50–1.21)	0.181
AA	32(10.3)	35(14.8)	1.41(0.75–2.46)	0.267
AA+GA vs. GG			0.93(0.59–1.40)	0.511
AA vs. GG+GA			1.59(0.86–2.71)	0.109
G	427(68.4)	319(67.6)	1.00(ref)	
A	197(31.6)	153(32.4)	1.11(0.82–1.47)	0.778
**rs6577555**
CC	196(62.8)	148(62.7)	1.00(ref)	
CA	99(31.7)	80(33.9)	1.11(0.69–1.72)	0.721
AA	17(5.5)	8(3.4)	0.68(0.31–1.62)	0.289
AA+CA vs. CC			1.02(0.71–1.60)	0.975
AA vs. CC+CA			0.70(0.29–1.76)	0.253
C	491(78.7)	376(79.6)	1.00(ref)	
A	133(21.3)	96(20.4)	0.97(0.72–1.38)	0.697


*^†^Adjusted by gender, age, smoker, diabetes and hypertension.*

### Linkage Disequilibrium and Haplotype Analysis

Linkage disequilibrium and haplotype analysis were also performed. As shown in Table 5, seven possible haplotypes were listed, the rs12128240 was in strong linkage disequilibrium with the rs2666433 (D′ = 0.904) and the rs6577555 (D′ = 0.846), and the rs2666433 was also in strong linkage disequilibrium with the rs6577555 (D′ = 0.801). Moreover, CGC and CAC were identified as the two main haplotypes in both controls (21.5 and 24.3%, respectively) and cases (37.6 and 36.6%, respectively). Nevertheless, we did not find a significant association between miR-34a haplotypes and the risk of IS.

**Table 5:** Haplotype analysis of the miR-34a polymorphisms with risk of IS.

Haplotype	Controls (2*n* = 1120)	IS patients (2*n* = 1096)	OR (95% CI)	*P*
CGC	420(37.6)	400(36.6)	1.00(ref)	
CAA	56(5.0)	48(4.3)	0.90(0.60–1.36)	0.641
CAC	241(21.5)	266(24.3)	1.16(0.93–1.45)	0.195
CGA	139(12.4)	128(11.6)	0.97(0.73–1.28)	0.811
TGA	31(2.7)	45(4.1)	1.52(0.95–2.46)	0.083
TGC	207(18.5)	175(15.9)	0.89(0.70–1.13)	0.337
TAC	26(2.3)	34(3.2)	1.37(0.81–2.33)	0.238


### Association Between the rs2666433 Polymorphism and the Expression Level of miR-34a

**FIGURE 1:**
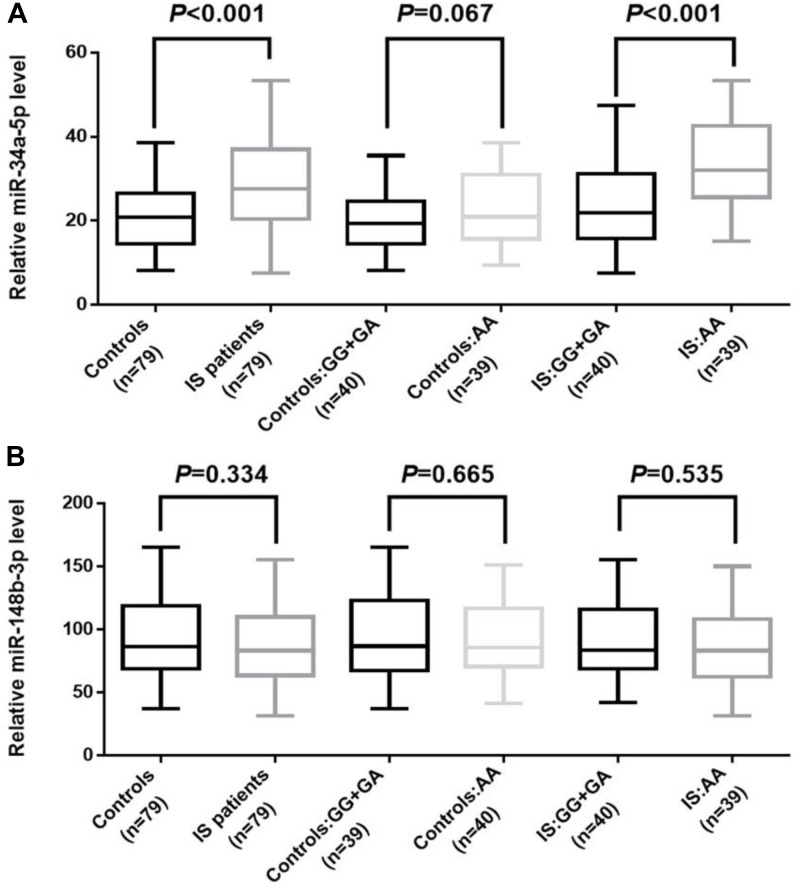
Association between rs2666433 polymorphism and expression level of miR-34a. The effect of rs2666433 polymorphism on the expression of miR-34a in controls and IS patients. **(A)** Increased level of miR-34a in IS patients compared to controls (*P* < 0.001), especially in IS patients carrying the rs2666433 AA compared with those carrying the rs2666433 GG+GA (*P* < 0.001). **(B)** As a negative control, plasma levels of miR-148b showed no significant differences among IS patients, controls and different genotype groups (*P* > 0.05). Plasma levels of miRNAs were normalized to cel-miR-39. The upper and lower boundaries of the boxes represent the 25th and 75th percentiles of the expression value. The upper and lower whiskers represent the maximum and minimum values. The horizontal line within the box represents the median value. All *P-*values were adjusted by gender, age, smoker, diabetes and hypertension.

The plasma levels of miR-34a and miR-148b were examined among the IS patients and the controls. The average RNA integrity number among samples was 8.6. As shown in Figure 1, the plasma level of miR-34a was significantly up-regulated in IS patients compared with the controls (*P* < 0.001). Particularly, we found that patients with AA genotype of rs2666433 had a higher level of miR-34a than those with GG+GA genotypes (*P* < 0.001). Controls with AA genotype of rs2666433 also had a higher level of miR-34a than those with GG+GA genotypes, but the difference was not significant (*P* = 0.067). As a negative control, the plasma level of miR-148b showed no significant differences among IS patients, controls and different genotype groups (*P* > 0.05). The clinical characteristics and genotypes distribution of qPCR subgroups are shown in Supplementary Table 4.

### Association Between the rs2666433 Polymorphism and the Serum Level of Homocysteine

**FIGURE 2:**
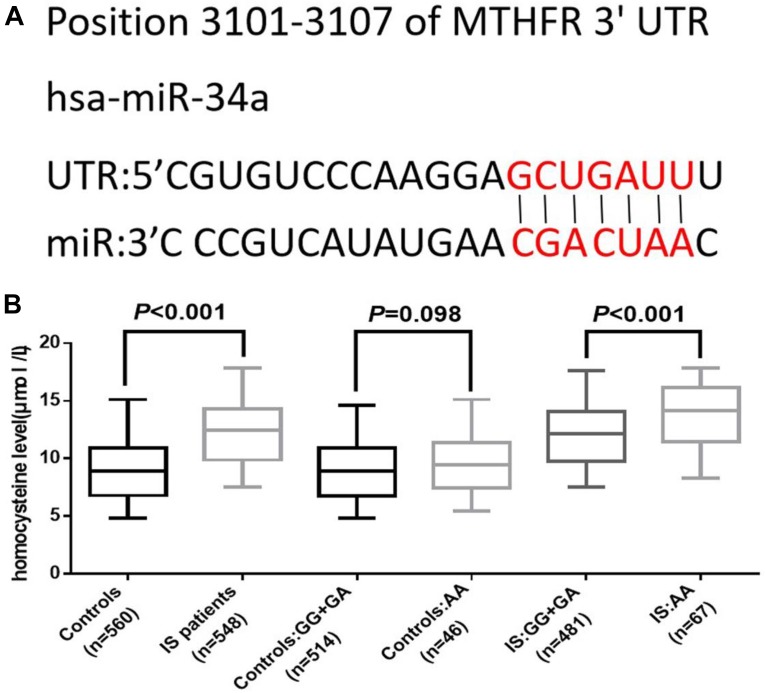
Association between rs2666433 polymorphism and serum level of homocysteine. The effect of rs2666433 polymorphism on the serum level of homocysteine in controls and IS patients. **(A)** Bioinformatics software predicted that MTHFR gene was a target of miR-34a. **(B)** Increased level of homocysteine in IS patients compared to controls (*P* < 0.001), especially in IS patients carrying the rs2666433AA compared to those carrying the rs2666433 GG+GA (*P* < 0.001). The upper and lower boundaries of the boxes represent the 25th and 75th percentiles of the homocysteine value. The upper and lower whiskers represent the maximum and minimum values. The horizontal line within the box represents the median value. MTHFR: methylenetetrahydrofolate reductase; UTR: untranslated region.

We further analyzed the association between rs2666433 polymorphism and serum level of homocysteine. As shown in Figure 2B, the serum level of homocysteine was significantly up-regulated in IS patients compared with the controls (*P* < 0.001). Additionally, after correlation analysis of the rs2666433 polymorphism and the serum level of homocysteine, we found that patients with the rs2666433 AA genotype had a higher level of homocysteine than those with GG+GA genotypes (*P* < 0.001). Controls with AA genotype of rs2666433 also had a higher level of homocysteine than those with GG+GA genotypes, but the difference was not significant (*P* = 0.098). As shown in Figure 2A, after predicting the potential targets of miR-34a by software Targetscan, Methylenetetrahydrofolate reductase (MTHFR) was found to be a potential target of miR-34a. The relevant information of bioinformatics analysis is shown in Supplementary Table 5.

### Genotypes and Alleles Distribution of rs2666433 in Different Populations

Considering the importance of rs2666433 polymorphism in the pathogenesis of IS, we further compared the genotypes and alleles distribution of rs2666433 in 11 different populations. As shown in Table 6, the genotypes and alleles distribution of rs2666433 in the present study were significantly different from 1KGP-ESN, 1KGP-ASW, 1KGP-CLM, 1KGP-PUR, 1KGP-TSI, 1KGP-CEU, and 1KGP-PJL populations (*P* < 0.05). Nevertheless, we did not find significant difference after comparing with 1KGP-CHS, 1KGP-CHB, and 1KGP- JPT populations (*P* > 0.05).

**Table 6:** Genotypes and alleles distribution of rs2666433 in different populations.

Populations	*n*	Genotypes (%)			*P*	Alleles (%)		*P*
		GG	GA	AA		G	A	
Present data	560	286(51.1)	228(40.7)	46(8.2)		800(71.4)	320(28.6)	
1KGP-CHS	105	56(53.3)	37(35.2)	12(11.4)	0.406	149(71.0)	61(29.0)	0.889
1KGP-CHB	103	53(51.5)	42(40.8)	8(7.8)	0.988	148(71.8)	58(28.2)	0.903
1KGP-JPT	104	58(55.8)	41(39.4)	5(4.8)	0.420	157(75.5)	51(24.5)	0.232
1KGP-ESN	99	20(20.2)	60(60.6)	19(19.2)	<0.001	100(50.5)	98(49.5)	**<0.001**
1KGP-ASW	61	18(29.5)	36(59.0)	7(11.5)	0.006	72(59.0)	50(41.0)	**0.004**
1KGP-CLM	94	66(70.2)	24(25.5)	4(4.3)	0.003	156(83.0)	32(17.0)	**0.001**
1KGP-PUR	104	78(75.0)	24(23.1)	2(1.9)	<0.001	180(86.5)	28(13.5)	**<0.001**
1KGP-TSI	107	89(83.2)	16(15.0)	2(1.9)	<0.001	194(90.7)	20(9.3)	**<0.001**
1KGP-CEU	99	83(83.8)	15(15.2)	1(1.0)	<0.001	181(91.4)	17(8.6)	**<0.001**
1KGP-PJL	96	64(66.7)	31(32.3)	1(1.0)	0.004	159(82.8)	33(17.2)	**0.001**


*1KGP: 1000 genomes project; CHS: Southern Han Chinese; CHB: Han Chinese in Beijing; JPT: Japanese in Tokyo, Japan; ESN: Esan in Nigeria; ASW: African Ancestry in Southwest United States; CLM: Colombian in Medellin, Colombia; PUR: Puerto Rican in Puerto Rico; TSI: Toscani in Italy; CEU: Utah residents with Northern and Western European ancestry; PJL: Punjabi in Lahore, Pakistan. *P* < 0.05 were indicated in bold font.*

## Discussion

To the best of our knowledge, this is the first study which investigated the association between rs12128240, rs2666433, and rs6577555 of the miR-34a gene and IS susceptibility. The main findings of the present study are listed as below: (I) MiR-34a rs2666433 polymorphism was associated with an increased risk of IS. (II) The level of miR-34a was significantly up-regulated in IS patients compared with the controls, and patients with rs2666433 AA genotype had a higher level of miR-34a than those with GG+GA genotypes. (III) Increased level of homocysteine was observed in IS patients compared to the controls, especially in patients carrying the rs2666433AA genotype compared to those carrying the rs2666433 GG+GA genotypes. Taken together, these findings indicate that the rs2666433 polymorphism may contribute to the susceptibility of IS.

In China, there are millions of people suffering from IS, which has brought great economic and health burdens to many families and society (Liu et al., 2011). Clinical and epidemiological studies suggested genetic factors played an important role in IS pathogenesis (Jerrard et al., 2003). As previously reported, miR-34a, a tumor suppressor miRNA in many species, suppresses proliferation and metastasis of tumor cells by silencing target mRNAs (Li R. et al., 2015; Li T. et al., 2015; Liang et al., 2015). In 2011, analysis of miRNAs expression profile performed by Raitoharju et al. (2011) revealed that miR-34a is upregulated in human atherosclerotic plaques. These results suggested the possibility of using miR-34a as a biomarker in atherosclerosis-related diseases such as IS. Subsequently, research conducted in 2015 showed overexpression of miRNA-34a in blood samples of acute IS patients and a rat model of middle cerebral occlusion compared to the controls (Liang and Lou, 2016). In contrast, inhibition of miR-34a prevented endothelial cell apoptosis in the setting of atherosclerosis (Li et al., 2018). In this study, overexpression of miR-34a in IS patients was also observed, validating the results of previous studies. Taking these results together, miR-34a may be related to the etiology of IS, but not every individual with up-regulation of miR-34a eventually suffered from IS, indicating that genetic variants like SNPs may also be involved in the etiology of IS.

Single nucleotide polymorphisms located in miRNA have been widely reported as the cause of abnormal functions of the miRNAs. Previously, some studies have reported that SNPs located in the miR-34a gene were associated with susceptibility to multiple human diseases such as osteosarcoma (Lv et al., 2014), colon cancer (Jiang et al., 2017), type 2 diabetes (Sun et al., 2018) and IS (Choi et al., 2016). To date, few studies focused on the association between miR-34a gene polymorphisms and IS susceptibility. In this study, we failed to identify a positive association between the rs6577555 polymorphism and IS or IS subtypes. Consistent with our data, Choi et al. (2016) also could not find any rs6577555 polymorphism frequency differences between IS and control groups in a Korean population. It may be ascribed to a similar distribution of SNPs among different East Asian populations. Table 6 supported our assumption, since the genotypes and alleles distribution of rs2666433 in the Guangxi population was found to be similar to those in 1KGP-CHS, 1KGP-CHB and 1KGP- JPT populations. So as far as we know, this is the first report of the association between the rs12128240 or rs2666433 and IS risk. In the present study, it was found that the rs12128240 polymorphism is not related to IS or IS subtypes. There is one possible explanation for this result. Rs12128240 is a sequence variant locating in 3′ flanking region of the miR-34a gene, which lacks important gene regulation related elements. Promisingly, we found a positive association between the rs2666433 polymorphism and IS or IS subtypes. AA genotype of rs2666433 was associated with a significantly increased risk of IS and LAA subtype compared with GG genotype. Moreover, A allele was also associated with a significantly increased risk of LAA subtype compared with G allele. These results indicated that rs2666433 may be involved in the etiology of IS.

More and more studies revealed that SNPs of the gene promoter can modulate the promoter transcription activity and the gene expression level by affecting binding efficiency for transcriptional factors (Hamaoka et al., 2013; Ma et al., 2016). Additionally, using bioinformatics analysis, the rs2666433 polymorphism in the promoter may affect the binding of transcription factors (Sun et al., 2018). In consideration of the evidence, we deduced that rs2666433 may influence the expression level of miR-34a. Subsequently, we detected the expression level of miR-34a and assessed miR-34a expression with rs2666433 polymorphism. In the current study, the plasma level of miR-34a was significantly up-regulated in IS patients compared to controls. Additionally, patients with rs2666433 AA genotype had a higher level of miR-34a than those with GG+GA genotypes. These outcomes support our assumption.

Based on a number of previously published clinical evidence, a significantly increased homocysteine level has been regarded as a crucial role in the formation of atherosclerosis and IS (Sacco et al., 2004; Fang et al., 2014; Chen et al., 2015). MTHFR is a key enzyme which plays an important role in the metabolism of homocysteine. A lower expression level of MTHFR causes accumulation of homocysteine, and then results in a higher level of homocysteine (Moll and Varga, 2015; He W. et al., 2017; Liu et al., 2018). According to research conducted by Zhang et al. (2015), as a member of the miR-34 family, miR-34b may influence the serum level of homocysteine by binding the 3′-UTR of MTHFR mRNA. After predicting the potential targets of miR-34a by software Targetscan, we found that MTHFR was also a potential target of miR-34a. Inspired by the observations above, we investigated the association between miR-34a rs2666433 polymorphism and serum level of homocysteine. In the present study, the serum level of homocysteine was significantly up-regulated in IS patients, and patients with the rs2666433 AA genotype had a significantly higher level of homocysteine than those with GG+GA genotypes. All the results above suggested that miR-34a rs2666433 AA genotype might upregulate the level of miR-34a, which in turn downregulated the level of MTHFR, resulted in the accumulation of homocysteine and IS occurrence.

In this study, controls with AA genotype of rs2666433 also had higher levels of homocysteine and miR-34a than those with GG+GA genotypes, but the difference was not significant. There was an explanation for this phenomenon. As we know, the miRNA expression can be regulated by the polymorphism in the promoter of a gene. Conversely, the polymorphism in the promoter of a gene is not the only factor that could affect the miRNA expression. Many studies reported that the long non-coding RNAs including Lnc-MEG3 (Huang et al., 2017a) and Lnc-OC1 (Tao et al., 2018), circular RNAs including circ-GFRA1 (He R. et al., 2017) and circ-NFIX (Xu et al., 2018) and coding RNA including PDL1 (Huang et al., 2017b) and LDHA (Xiao et al., 2016), can function as competing endogenous RNAs to regulate miR-34a expression by sponging miR-34a. Under normal conditions, the normal expression of these genes does not affect the expression of miR-34a. However, under pathological conditions, down-regulation of these genes can further up-regulate the expression of miR-34a based on the up-regulatory effect of the miR-34a polymorphism. Therefore, a gene-gene interaction analysis in the future will better reveal the roles of miR-34a and its regulatory genes in the etiology of ischemic stroke.

In the present study, we also investigated the association between the miR-34a polymorphisms and IS severity. However, no significant association was found between the miR-34a polymorphisms and IS severity. Additionally, 7 possible haplotypes were found in our study, and CGC and CAC were the two main haplotypes in both controls and cases. Nevertheless, we did not find a significant association between miR-34a haplotypes and the risk of IS.

There are some limitations in the current study. First, our results are just representative for IS patients of Guangxi Chinese in southwest China, data from other patient cohorts is also needed to verify our results. Second, because of selection bias, the results may be influenced by control and case ascertainment. Third, it should be acknowledged that the differences in characteristics among qPCR subgroups may have a potential effect on the expression profile of miR-34a.

In summary, the present study found the association between miR-34a polymorphisms and the risk of IS in the Guangxi Chinese in southwest China. The results may provide some evidence for etiology of IS and a potential biomarker or therapeutic target for IS. In order to validate the results, further studies in ethnically disparate populations are going to be carried out.

## Ethics Statement

The study got the approval by the Ethics Committee of Youjiang Medical College, and written informed consent was obtained from all patients and controls prior to entering the study.

## Author Contributions

G-JW and M-QY wrote the manuscript. G-JW, M-QY, L-HJ, Y-LL, C-HL, H-CL, and H-TH collected samples and performed the experiments. Z-QQ and Y-SW conceived and designed the experiments.

## Conflict of Interest Statement

The authors declare that the research was conducted in the absence of any commercial or financial relationships that could be construed as a potential conflict of interest.

## Footnotes

1 http://www.ncbi.nlm.nih.gov/SNP

2 http://www.internationalgenome.org

3 http://www.targetscan.org/mamm_31/
