# Supplementary material for: A Genetic Variant of miR-34a Contributes to Susceptibility of Ischemic Stroke Among Chinese Population
Source: Front Physiol. 2019 Apr 24;10:432. doi: 10.3389/fphys.2019.00432 (PMC6491571; doi:10.3389/fphys.2019.00432)
Supplement: Supplementary file 1 [file Table_1.docx]

**Supplementary Table 1. Primers for genotyping SNPs in miR-34a**

| **SNP loci** | **Sequences of PCR primers** | | **Sequences of extension primers** |
| --- | --- | --- | --- |
| rs12128240 | Upstream primer | 5'-CATCAGCCACTGGAGCAGAGAA-3' | 5'-TTTTTTTTGAATCTGCGCCCAACCACC-3' |
|  | Downstream primer | 5'-AGGTTTTGAACGCGAGCAATCT-3' |  |
| rs2666433 | Upstream primer | 5'-TGCCAGGCCAAGATCTAGTGAC-3' | 5'-TTTTTTGGGTGTGGGGGCATGAAGG-3' |
|  | Downstream primer | 5'-CTCTTTCCCCCTTGCACAGAGA-3' |  |
| rs6577555 | Upstream primer | 5'-GGAGGATCACTTGAGGCGAGAA-3' | 5'-TTTTTTTTTTTTTTTGTAGAGACAGTTGCTGAAGGTGG-3' |
|  | Downstream primer | 5'-ATTGCTTCCCTTTCACGCAGAC-3' |  |

**Supplementary Table 2. Clinical characteristics of LAA and SAO subgroups**

| **Variables** | **LAA (n=207)** | **SAO (n=341)** | ***P* ^†^** |
| --- | --- | --- | --- |
| Gender (M/F) | 133/74 | 220/121 | 0.950 |
| Age, years (mean±SD) | 58.92±9.44 | 59.59±8.99 | 0.273 |
| Smoker, n (%) | 62(30.0) | 96(28.1) | 0.652 |
| Hypertension, n (%) | 109(52.6) | 182(53.4) | 0.941 |
| Diabetes mellitus, n (%) | 63(30.4) | 95(27.7) | 0.519 |

IS: ischemic stroke; SD: standard deviation; M: male; F: female; LAA: large artery atherosclerosis; SAO: small artery occlusion.

†Two-sided chi-square test or student’s t-test.

**Supplementary Table 3. Clinical characteristics of NHISS<6 and NHISS**≥**6 subgroups**

| **Variables** | **NHISS<6 (n=312)** | **NHISS**≥**6 (n=236)** | ***P* ^†^** |
| --- | --- | --- | --- |
| Gender (M/F) | 200/121 | 153/83 | 0.541 |
| Age, years (mean±SD) | 58.97±8.95 | 59.81±11.03 | 0.192 |
| Smoker, n (%) | 82(26.3) | 76(32.2) | 0.130 |
| Hypertension, n (%) | 151(48.4) | 140(59.3) | **0.011** |
| Diabetes mellitus, n (%) | 76(24.4) | 82(34.7) | **0.008** |

IS: ischemic stroke; SD: standard deviation; M: male; F: female.

†Two-sided chi-square test or student’s t-test.

*P* < 0.05 were indicated in bold font.

**Supplementary Table 4. Clinical characteristics and genotypes distribution of qPCR subgroups**

| **Variables** | **Controls (n=79)** | **IS patients (n=79)** | ***P* ^†^** |
| --- | --- | --- | --- |
| Gender (M/F) | 55/24 | 51/28 | 0.498 |
| Age, years (mean±SD) | 60.36±10.73 | 59.92±10.11 | 0.296 |
| Smoker, n (%) | 14(17.7) | 23(29.1) | 0.091 |
| Hypertension, n (%) | 17(21.4) | 37(46.8) | **<0.001** |
| Diabetes mellitus, n (%) | 9(11.4) | 27(34.2) | **<0.001** |
| GG+GA | 40(50.6) | 40(50.6) |  |
| AA | 39(49.4) | 39(49.4) | 1.000 |

IS: ischemic stroke; SD: standard deviation; M: male; F: female.

†Two-sided chi-square test or student’s t-test.

*P* < 0.05 were indicated in bold font.

**Supplementary Table 5. Relevant information of bioinformatics analysis**

| **miRNA** | **predicted target** | **predicted target site** | **Context++ score percentile** |
| --- | --- | --- | --- |
| has-miR-34a | MTHFR | 3101-3107 | 57 |
|  | KLF2 | 3653-3659 | 60 |
|  | GPR113 | 851-857 | 40 |
|  | CNTNAP3 | 3211-3217 | 45 |
|  | PCDHB5 | 2910-2916 | 57 |

KLF2: kruppel-like factor 2; GPR113: G protein-coupled receptor 113; CNTNAP3: contactin associated protein-like 3; PCDHB5: protocadherin beta 5.

**Supplementary Figure 1. Sequencing diagrams of miR-34a SNPs.**
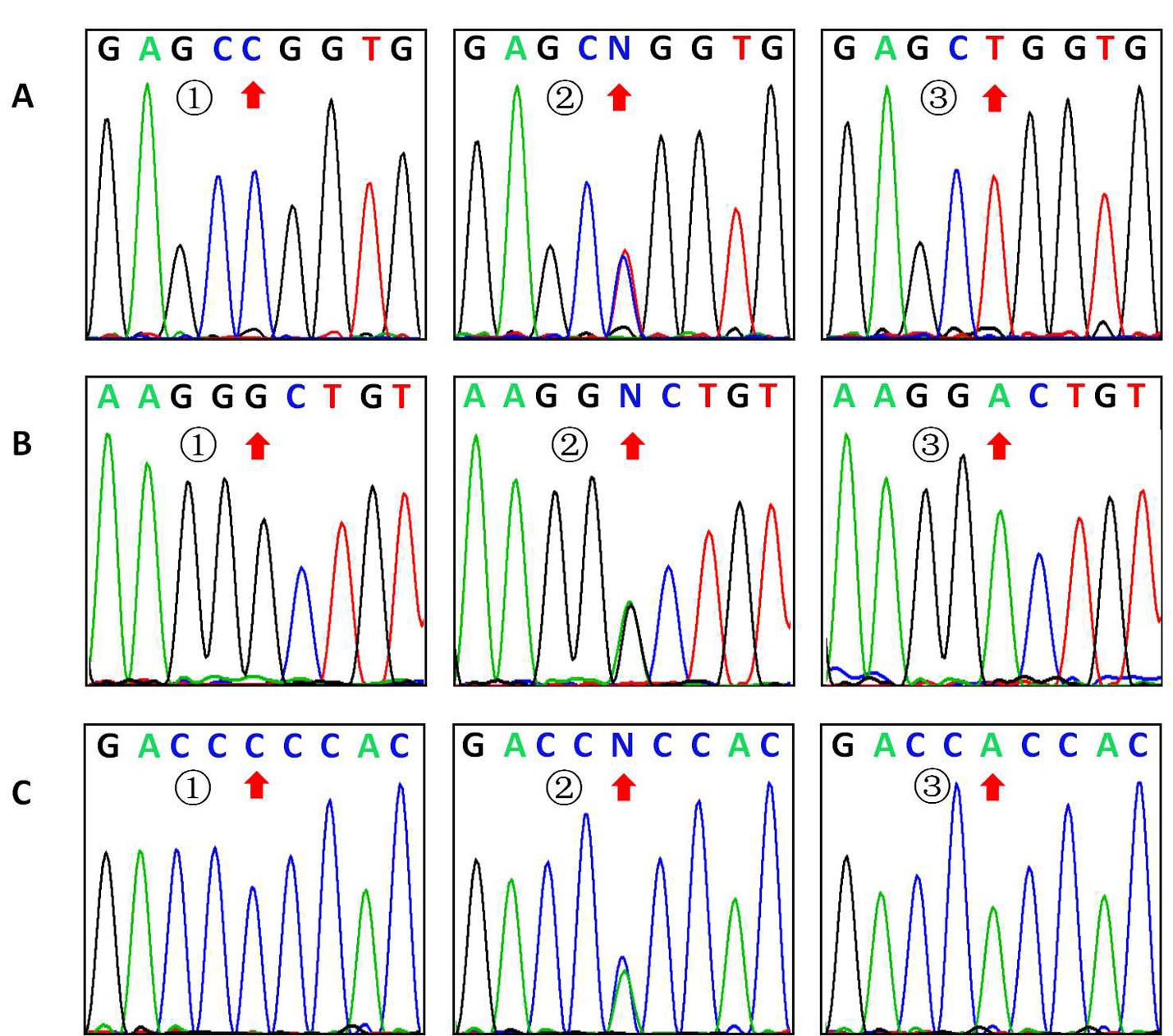


(**A**) The numbers ①, ② and ③ represent CC, CT and TT genotypes for rs12128240 respectively. (**B**) The numbers ①, ② and ③ represent GG, GA and AA genotypes for rs2666433 respectively. (**C**) The numbers ①, ② and ③ represent CC, CA and AA genotypes for rs6577555 respectively.
